# Clean manufacturing powered by biology: how Amyris has deployed technology and aims to do it better

**DOI:** 10.1007/s10295-020-02314-3

**Published:** 2020-10-07

**Authors:** Paul Hill, Kirsten Benjamin, Binita Bhattacharjee, Fernando Garcia, Joshua Leng, Chi-Li Liu, Abhishek Murarka, Douglas Pitera, Elisa Maria Rodriguez Porcel, Iris da Silva, Chuck Kraft

**Affiliations:** 1 Amyris, Inc., 5885 Hollis Street, Ste. 100, Emeryville, CA 94608 USA; 2 Amyris Brasil Ltda., Rua John Dalton, 301-Bloco B-Edificio 3, Condominio Techno Plaza, Campinas, SP 13069-330 Brazil

**Keywords:** Technology transfer, Fermentation, Farnesene, Scale-up, Amyris

## Abstract

Amyris is a fermentation product company that leverages synthetic biology and has been bringing novel fermentation products to the market since 2009. Driven by breakthroughs in genome editing, strain construction and testing, analytics, automation, data science, and process development, Amyris has commercialized nine separate fermentation products over the last decade. This has been accomplished by partnering with the teams at 17 different manufacturing sites around the world. This paper begins with the technology that drives Amyris, describes some key lessons learned from early scale-up experiences, and summarizes the technology transfer procedures and systems that have been built to enable moving more products to market faster. Finally, the breadth of the Amyris product portfolio continues to expand; thus the steps being taken to overcome current challenges (e.g. automated strain engineering can now outpace the rest of the product commercialization timeline) are described.

## Introduction

Amyris is a fermentation product company that leverages synthetic biology and has been bringing novel fermentation products to the market since 2009. Founded in 2003 to create a reliable supply of cost-effective artemisinin for the treatment of malaria, Amyris has now scaled up and manufactured nine distinct fermentation molecules (terpenoids like farnesene, manool, bisabolol; terpenoid-based species like RebM; and non-terpenoid products) with multiple contract manufacturing organization (CMO) partners around the world. The foundation of that achievement is an investment of > $500 M in building powerful Research and Development (R&D) capabilities—essentially industrializing the design/build/test paradigm, enabling orders of magnitude increases in the rate of strain generation and testing, significantly improving the quality of the strain construction, and building data systems to accelerate learning.

Additionally, significant technical innovations have helped to ensure that these strains succeed in the commercial fermentation environment. For example, Amyris strain engineers have pioneered the reprogramming of central metabolism to significantly improve product yields and metabolic rates [1]. Simple, proprietary, and robust genetic switches have been developed which preserve cellular productivity for weeks in fermentation culture [2]. And the power of the high-throughput screening apparatus has enabled efficient enzyme discovery and engineering, significantly improving the catalytic rates of multiple independent enzymes in each new biosynthetic pathway, in some cases improving the catalytic rate tenfold or more. For each of the nine products taken to industrial scale, at least one enzyme in the heterologous pathway has been improved via mutagenesis. Therefore, for the process development (PD) scientists and engineers, partnering with this team has created multiple challenging opportunities to develop unique fermentation and downstream purification processes and to scale them up to industrial scale.

New small molecule targets are being made now by fermentation at costs low enough to enable profitable production of whole classes of new molecules. In the past decade, Amyris has completed more than thirty successful technology transfers to ten different full-scale fermentation manufacturing facilities on three continents. The molecules, both liquids and solids at fermentation temperatures, have then been purified at eight distinct downstream purification (DSP) facilities. Much has been learned about both what it takes to enable these processes to work well at scale and about the best practices undertaken to deploy that technology.

Process development and manufacturing organizations will increasingly find themselves put under increased pressure to optimize resource allocation as the pace of new product development will likely quicken. These groups need to think deeply about their deployment strategy to ensure they adopt a balanced and effective approach that matches the start-up challenges to the risk tolerance of the organization.

## Key aspects of a successful process deployment strategy

Why does efficient technology transfer matter? One answer to this question is to estimate the financial consequences of losing one week of production in a sold-out facility due to a technology transfer failure. For a product selling for $15/kg in a facility with 6 × 200 m^3^ fermentors, one lost week of production could easily be $1.5 M in lost revenue.^1^ Further, when the impacts on the business relationships (with partners or customers) and the team’s morale are considered, being successful as quickly as possible has great value. Therefore, investments in maximizing the odds of success in rapid technology adoption can often pay for themselves.

Much has been written about successful approaches to technology transfer. Many of these describe either the high-level process of technology transfer [3–6]—that is, the organization and systems utilized for success—or primarily focus on the technical aspects of scale up or technology transfer [7–12], several wisely adopting a “Begin with the End in Mind” approach consistent with Stephen Covey’s habits [13]. Few previous authors have described an approach to balancing the technology development and technology transfer resource optimization in the age of the synthetic biology revolution, perhaps because until recently, it has been uncommon for organizations to routinely face the challenge of delivering multiple new fermentation-derived molecules to manufacturing every year.

In the sections below, key aspects of these references will be folded into the lessons learned at Amyris over the past decade to articulate the key elements of managing risks in technology transfer; namely, developing the process with the end in mind, adopting the right technology transfer philosophy, and committing the right level of resourcing to the technology transfer.

### Develop the process with the “End in Mind”

#### At lab scale, implement scaled-down process models of the targeted facility

Implementing the techniques for scaling down full-scale process conditions to the lab or pilot scale is a critical aspect of a successful technology transfer approach. However, the best representation of the full-scale environment at lab scale can be onerous and resource intensive to implement routinely. Therefore, to succeed in fermentation development and technology transfer, a tiered approach to experimental development can be adopted, in which each successive tier becomes lower in throughput but higher in fidelity to the full-scale environment. For example, in a first tier that is focused on fermentations to evaluate and rank strains, shorter inoculum development procedures could be used, but with the same raw materials and feeding algorithms as will be used in the manufacturing plant. At the next experimental tier, the critical aspects of the large-scale fermentation environment are built-in, including:

- matching inoculum cumulative population doublings and conditions, including a final seed fermentor stage, to better match the full-scale inoculum qualities.
- matching average temperature, pH, and OTR conditions; as is appropriate and possible, match the air sparge vessel volumes per minute and/or average dissolved gas partial pressure.
- utilizing the same feed sugar sterilization approach.

Fermentations in this second tier constitute the vast majority of the scaled-down fermentations done in the lab or pilot scale. Lastly, to minimize scale-up risk, additional experiments (tier 3) are added as needed to model some important aspects of the full-scale environment and process control variability which can be too complex to routinely implement:

- the impact of dissolved gas heterogeneities: pO_2_, pCO_2_;
- the potential for increased foam formation—experiments to match the superficial gas velocity and/or gas disengagement zone conditions;
- the impact of liquid phase heterogeneity due to longer mixing times at commercial scale;
- the effect of utility outages leading to process interruptions.

**Fig. 1:**
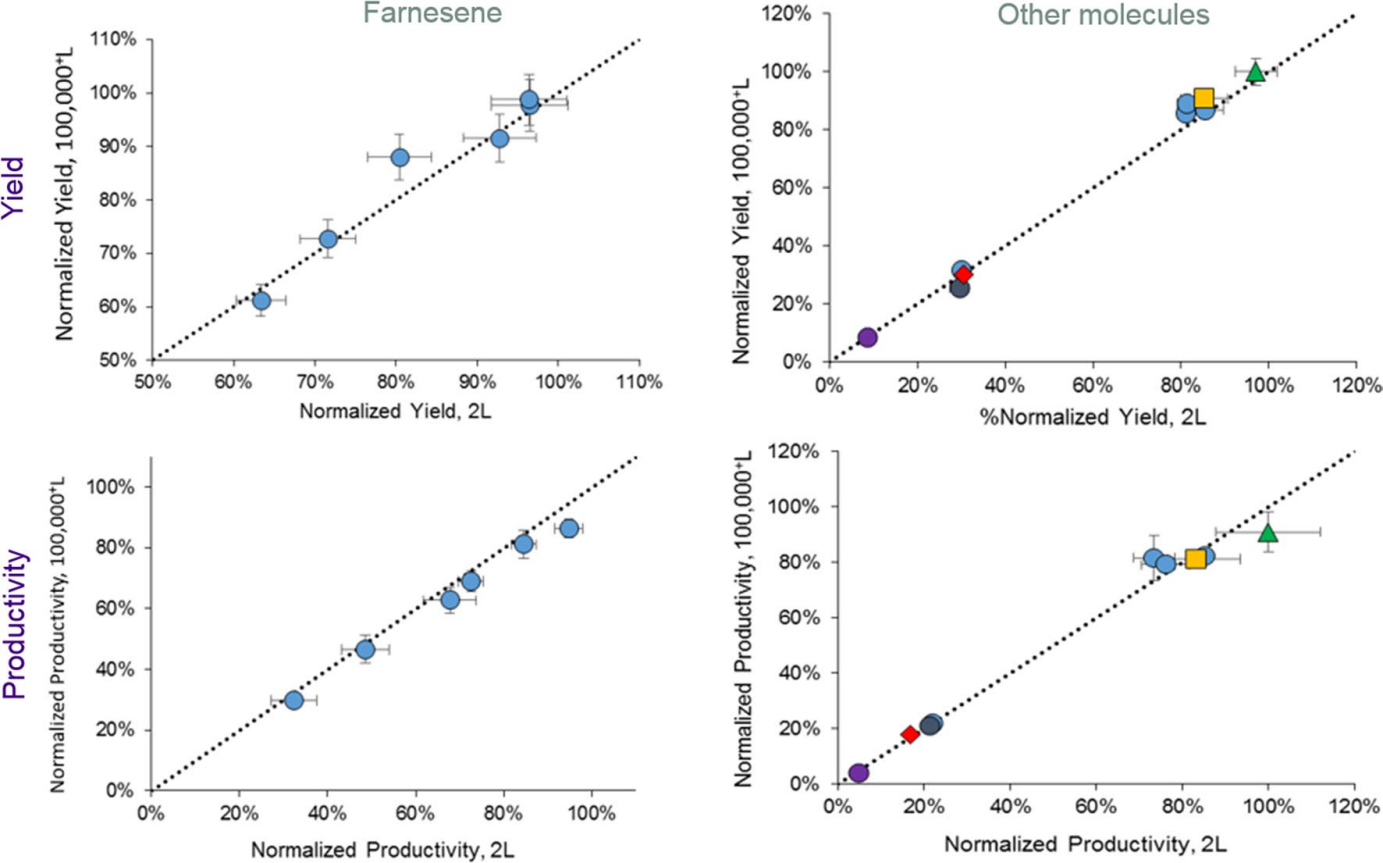
Lab-scale fermentation performance routinely predicts 100^+^ m^3^ performance. Different colors/shapes represent different products, and the values are normalized to the maximum value in each set. There is a wide spread in average selling price amongst the different products; thus, the fermentation performance enabling commercial success is achieved at very different fermentation yields and productivities (color figure online)

Implementing the approach above has helped Amyris build a good track record of fermentation scale-up success to both bubble columns and stirred tank reactors. For multiple fermentation technology transfers for multiple different products, as measured by fermentation product yield on sugar (g product/g sugar) or volumetric productivity (g product/L/h), Fig. 1 demonstrates that the averaged lab-scale, scaled-down fermentation performance is essentially equivalent to the performance of the same strain and process executed at commercial scale. This goes a long way to validate the lab-scale, scaled-down fermentation approach.

It is acknowledged that the majority of the Amyris fermentation technology transfers have been with *Saccharomyces cerevisiae*. Nevertheless, these data do suggest that to date (1) the stated approach to fermentation scale-down minimizes technical risk in the technology transfer for any organism and (2) the challenges associated with operating at the 100–250 m^3^ fermentation scale (e.g. environmental heterogeneity) do not appear to be significantly impacting the fermentation performance for the nine different molecules scaled to date.

Also, while it is not covered above, this same approach to modelling the large-scale fermentation environment at lab scale can and must be applied to downstream processing unit operations insofar as possible. For example, the majority of pilot-scale liquid–solid centrifugation development will use pilot-scale centrifugation equipment. Scaling the feed rates using sigma factor theory does a good job of predicting supernatant quality. But differences between the conditions in that pilot equipment and the full-scale equipment—such as shear in the inlet distributor or aspects of heavy phase solids handling—should be evaluated with additional experiments so as not to cause surprises when scaling up. Similarly, particular aspects of scaling evaporation, filtration, adsorption, etc. will require scale-up specific experimentation in addition to the more routine pilot experimentation.

Lastly, operations at full scale can take longer than they are typically scheduled to take at pilot scale. Understanding this and including experiments to test the impact of longer waiting or holding times (at the right temperature) in the scaled-down modelling is critical.

### Adopt a technology transfer philosophy to ensure the right focus

#### Organize technology transfer support well in advance with delineation of start-up roles and responsibilities and pre-work designed to speed troubleshooting

Excellence in the technical aspects of scale-up is insufficient to minimize risk of technology transfer failure. Many technology transfers struggle to succeed initially, not because the chemical engineering principles were poorly understood and applied, but because of the unexpected—the wrong hydration state of a critical raw material was ordered, or an unmonitored incubator could not consistently maintain the correct temperature. More generally, an important aspect of the process or process execution was not well communicated, was not double-checked, or was simply not under control.

Solving these unexpected challenges can take time and resources; thus, it is best to attempt to prevent these problems through a strategy of preparedness by creating a system to enable communication and enabling efficient and rapid troubleshooting in the start-up phase. That advance planning can extend all the way into a strategic delineation of roles and responsibilities and preparing action plans for possible failure modes prioritized by risk analysis.

A particularly good treatment of this approach to technology transfer is articulated in Dr. Charles Goochee’s discussion of the role of a Process Development group in a process start-up [4]. For brevity, only the key mantras of the start-up approach are repeated below. But perhaps the most important aspect of the entire approach is the buy-in by all participating groups that all parties are responsible for the success or failure of the process start-up. This contract can be critical to keep all parties actively engaged in the troubleshooting should something go awry in the first engineering runs. Given this, five *Mantras of Technology Transfer* are articulated, as follows:

1. *Manufacturing is the customer.*
  The manufacturing/operations team will own execution of the manufacturing process well after the initial start-up. They will be responsible for safely delivering the product on schedule and at the projected cost. Therefore, all groups must acknowledge that when technology is to be transferred, the Manufacturing group is the customer and ultimate decider of what can be implemented. The best chance for smooth process implementation is to employ process techniques that the Manufacturing group is familiar with. So, it is in the best interest of Process Development to accommodate the requests of the Manufacturing group, provided that process success is not put at risk.
  Further, the Manufacturing team has many responsibilities; for example, it should be the responsibility of the Operations team to ensure that the operations staff are well trained on the incoming process, that the facility receiving the process—the equipment, utilities, etc.—will perform as promised, and that any new equipment or facility modifications are completed in time for the first engineering runs.
2. *Process Development is responsible for the process.*
  In this division of focus, Process Development is responsible for defining the how, whens, whats, and whys of the process—essentially, the recipe for making the product. There are several critical aspects to this responsibility. Firstly, the PD team needs to design the process to fit the facility *as it is* (or will be) *built*, not as they wish or hope it will be. Because of that, the lab and pilot scaled-down process models must reflect this reality.
  Second, during the start-up, the Process Pevelopment team should make the process-specific decisions. The Manufacturing team will operate the equipment according to the batch records and standard operating procedures, but if a decision needs to be made as to the intent of the process definition, or if an intervention needs to be made to ensure proper process execution, the PD lead should have the final say, as they have built up the intuition and judgement about the process. This is only during the process start-up phase.
  To summarize the first two mantra:  it is in the best interest of process success for the Process Development group to accommodate the reasonable requests of the Manufacturing group. However, the Process Development team has ultimate accountability for process success. During the first manufacturing campaign, the Manufacturing group will have done its job if the equipment operates as advertised and the batch records are followed. If the process does not work because the manufacturing equipment does not operate as advertised or because batch records were not followed, the Manufacturing organization must take responsibility for these issues. On the other hand, if the equipment operates as intended and batch instructions are followed, and the process does not perform as expected, the Process Development group is responsible to have a plan in place to quickly identify the cause and corrective actions.
  PD is, therefore, also responsible for building and staffing a troubleshooting approach complete with a *Debug plan* for each unit operation at start-up. The Debug plan is a set of troubleshooting samples, data collection, and parallel experimentation that allows for rapid identification of the process problem source. One goal of the Debug Plan is to collect extra information and samples for analysis in advance to prevent the worst-case scenario: needing to begin another batch, also likely to fail, to collect the right samples and data to isolate the problem with the process.
3. *We do not do experiments in the plant (experimentation happens at lab and pilot scale).*
  Simply put, if a new process condition is attempted during the start-up of the full-scale process that has not been demonstrated at the lab or pilot scale, it is an experiment. Should the process performance of that step not meet expectations in the start-up run, the team will be left with uncertainty about whether the failure was a result of the new process condition or if there was some other scale-up issue. The cost of experimentation is much lower at the lab or pilot scale; thus, during start-up, only process conditions that have been validated should be attempted.
  It may seem obvious, but it is worth stating that a corollary to this mantra is that the overall technology transfer can be de-risked by executing a technology transfer to a second site—presumably at pilot scale—prior to the commercial-scale start-up. Demonstrating that the technology functions as expected at another site with another team helps to confirm the robustness of the process and the technology transfer approach and thus helps minimize the temptation to make modifications at full scale.
4. *Avoid “Pet Hypotheses” during problem resolution.*
  Should a start-up issue arise, troubleshooting benefits from a team-wide, comprehensive approach to ensure the completeness of the response. The authors are aware of technology transfers elsewhere in which, because of the incomplete nature of the hypothesis generation and the lack of parallel avenues of inquiry, a facility languished for more than a month until the team was able to identify the problem’s source. Including many, varied voices in the analysis, prioritizing, and pursuing multiple options is always the surest path to finding the best solution quickly.
5. *Use risk assessments to collect, analyze, and disseminate the right data.*
  In addition to the top four mantras articulated by Goochee [4], a fifth mantra is added to reflect the importance of developing an information collection plan guided by a risk assessment well in advance of the start-up by involving a diverse project team. Some benefits are listed below:
6. It can help ensure that the facility has the right sensors and the Quality team has the right analytical capabilities to produce the critical data and analyses at the optimal frequency. Note that the collection frequency might be different during start-up than for routine operations.
7. The possible failure modes captured in the risk assessment can shed light on what aspects of a start-up debugging plan should be emphasized, potentially compensating for a gap in established practices.
8. Finally, building a system for real-time comparison of actual vs. projected results and automated critical data analyses can help minimize the time to implement a useful intervention.

Additionally, the more that strategic data collection and analyses can be done and securely shared internally, the greater the potential contributions of expert personnel who are working far from the plant. As we have observed in 2020, world events and travel restrictions may interfere with in-person tech transfer support, often unexpectedly.

By adhering to the tenets above, Amyris has increased the probability of process transfer success in the first manufacturing batches in the plant (Table 2). However, we have not always chosen to staff start-up efforts to be able to maximize our ability to catch and quickly solve problems. This relates to the last aspect of the deployment strategy and acknowledges the resource constraints that every organization faces.

### Commit the right level of resources to the technology transfer

#### Tailor the level of PD support to the risk tolerance of your organization

Dr. Goochee’s recommendation [4] is that, for a typical fermentation process coupled with a three to five step DSP process, one dozen process development staff would be needed for the 6 months prior to the start-up plus ~ 1 month for start-up. These staff would not be focused on developing the process; rather, that group would be committed to the process of a successful technology transfer.

This kind of effort is not without cost. Completing most de-risking activities will minimize the calendar time needed to achieve the targeted plant performance but could cost the organization on the order of two million dollars or more^2^—a big commitment to technology transfer in the industrial microbiology space. Thus, a balancing of this strategy may be appropriate to match the risk tolerance of the organization to the effort associated with the technology transfer.

Amyris has executed over 30 technology transfers in the previous decade, and some aspects of those transfers naturally reduced the failure risk:

- Platform fermentation and downstream purification processes (in which only small details change from product to product) were developed where possible, minimizing product to product complexity at the manufacturing site.
- The same product is often produced at multiple sites (in part as a best practice for business continuity), building a good understanding of what is needed to support process performance at a new site.
- New products were introduced at previously used manufacturing locations to build on the trust, communication, and facility understanding of previously transfers.

However, as the product portfolio has broadened, so too has the variety in required individual DSP processes, forcing an expansion of the number of manufacturing groups since not all have the same DSP capabilities.

Based on these assessments, over time, the effort associated with technology transfer per product has been reduced, though admittedly with some increased risk. To optimize resources, we strive to match the risk associated with process transfer complexity to the magnitude of the effort in scale-up/start-up support (Table 1).

**Table 1:** Process complexity and risk should dictate the intensity of the start-up support

Relative technology transfer complexity	Risk score^a^	Support description	In-plant support
Same product, new strain, similar process, same site	0	“Light”	1 person on site for the first run
New product, similar process, same site	1	“Lean”	2–3 people on site. Minimal Debug Plan
New product, modified process, same site	2	“Moderate”	1 person per unit operation. Critical aspects of Debug Plan implemented
New product, modified process, new unit operations or new CMO	3	“Heavy”	1–2 people per new unit operation. Full Debug Plan. Specialized or vendor support on new unit Ops
New product, modified process, new unit operations, new CMOs	4	“Full Court Press”	Two support staff per unit operation. Full Debug lan including parallel fermentations and parallel DSP operations. Specialized or vendor support on new unit Ops

^a^Risk increases if the product is new, the process is new, there are new unit operations, and/or the manufacturing site is new

Since the successful start-up of the third fermentation molecule in 2013, 21 technology transfers to full-scale manufacturing have been completed, and the vast majority of them have been successful as judged by the relatively few number of batches needed to meet the technology transfer expectations (Table 2). Again, by adhering to the deployment philosophy mantras above as is possible, and executing the lab and pilot experimentation appropriately, the risks associated with optimized staffing can be minimized.

**Table 2:** Amyris fermentation technology transfer results for the past 8 years

Transfer #	Product	Prior facility experience?	New process?	Risk score	Intensity of TT support effort	# of batches needed to meet expectations
1	1	No	Yes	2	Full Court Press	2
2	3	No	Yes	3	Lean	~ 3
3	1	Yes	Yes	1	Lean	Never (tried 2)
4	1	Yes	Yes	1	Moderate	1
5	3	Yes	No	0	Moderate	2
6	1	Yes	No	0	Lean	1
7	4	Yes	Yes	2	Heavy	4
8	1	Yes	No	0	Lean	1
9	1	Yes	Yes	1	Moderate	8^a^
10	5	No	No	2	Moderate	2
11	6	Yes	No	1	Lean	1
12	3	Yes	No	0	Lean	1
13	1	Yes	No	0	Lean	5
14	5	Yes	Yes	1	Lean	1
15	6	Yes	Yes	1	Lean	1
16	3	Yes	Yes	1	Lean	1
17	7	Yes	Yes	2	Heavy	2
18	7	Yes	Yes	1	Heavy	2
19	8	Yes	No	1	Lean	1
20	9	No	Yes	3	Lean	1
21	7	Yes	Yes	1	Heavy	1

^a^90% of target performance was routinely achieved after three runs. Product #2 was scaled up prior to 2012. At some contract manufacturers, the participation by Amyris staff was limited due to CMO rules (i.e. for Product 9). Additionally, fermentation runs that failed due to equipment failure or contamination are not counted in tallying the number of batches needed to meet expectations

One last thought on technology transfer staffing: a standard practice when executing technology transfer is to complete one to two ‘engineering runs’ ahead of the full production campaign. These runs give the operations team some time to fully learn the new process and optimize equipment operation before committing to delivering product mass. However, in today’s reality, with increased time and cost pressures to deliver product to customers and in which product delivery cycles continue to shrink, it may be that organizations begin to count on delivering saleable materials from the very first engineering runs. Further, it is worth mentioning that in contrast to some other companies, Amyris often adopts a Go To Market strategy that prioritizes minimizing the time to the market (to seed the market and ramp demand), sometimes at the expense of maximizing the margin in the first manufacturing campaign. These factors clearly carry additional risk; thus, an increased resourcing commitment to aggressive debugging is warranted insofar as is possible.

The following case studies help illustrate some of the challenges faced in moving toward a risk-based staffing approach for resourcing of technology transfer support. The first case study describes one of the technology transfers completed as Amyris began to implement this approach, and the subsequent two highlight the challenges associated with this approach as applied to novel products or implementation of ‘minor’ changes.

## Case studies

### Case study #1: New farnesene strain and new fermentation process

The Brotas, Brazil manufacturing facility was opened at the end of 2012, and the start-up of the farnesene fermentation and recovery technology was executed with full process development support (Mantra #2: Process Development is responsible for the Process), including:

- 24h coverage by PD staff in the plant;
- Full Debug Plans were implemented, including collecting extra samples and data;
- PD staff executed parallel, co-located two-liter fermentors, allowing for rapid troubleshooting.

After some initial equipment challenges were overcome, the full-scale fermentors performed as was expected based on lab and pilot scale performance, validating the successful technology transfer. Subsequent transfers of new strain and process technology also went well, leading to the deeper consideration of the trade-offs in technology transfer resourcing as discussed above. The portfolio of products on which Amyris was working and taking to scale was expanding at the same time, putting more pressure on resourcing, and thus we began to strategically attenuate technology transfer resourcing. Specifically, in the subsequent farnesene technology transfers, we shifted support from “Full Court Press” to “Lean”, eliminating most on-site parallel experimentation.

In 2014, a farnesene strain with substantially higher lab-scale production performance was identified in the laboratory. However, the fermentation with this strain was described by the process development staff as being ‘finicky’ at lab scale: specifically, at low frequency, at variable culture run times, the fermentation performance would plummet, and the culture would essentially stall or ‘pout’, requiring an early harvest. Complicating matters further, to meet the available manufacturing window in the facility, the team bypassed some of the typical development checkpoints. For example, it was typically required that new strain and fermentation technology be demonstrated in the Brazilian pilot plant as a way of providing a double-check of the technology as well as an opportunity for training the manufacturing staff prior to start-up in the Brotas facility. This step was skipped to maintain the timeline.

Hence, while there was much excitement about the prospect of realizing significantly higher performance at full scale, there was more nervousness than usual on the technology transfer team. Was the finicky strain, coupled with the less complete technology transfer process and lighter debugging plan, good enough to transfer? After executing an informal risk assessment, the team was in part comforted by the fact that that technology transfer was going to a facility that had made the product before, so the staff there had familiarity with the general process. Further, it was recognized that the facility could revert to the previous strain and fermentation approach; thus, unlike many other technology transfers, there was a built-in hedge against the costs associated with a technology transfer failure.

At start-up, the new strain and fermentation technology was initiated in two of the six production fermentors (while the other four continued with the previous technology). In the last seed fermentor stage for the new strain, there were signs of struggle—excess ethanol accumulation and poorer culture growth. After inoculating the production fermentors, both cultures stalled, grew very poorly or not at all, which was a significant disappointment.

The initial troubleshooting response was to double-check everything: raw materials, set points, etc., but no difference in process execution was uncovered. The absence of parallel troubleshooting experiments prevented the rapid identification of the right area of concern. Thus, there existed a large matrix of possible culprits and the technology transfer team had to significantly ramp up efforts and call in additional resources while the manufacturing team resumed operations with the previous technology.

A multidisciplinary group was engaged to consider all options (Mantra #4: no pet theories). Within days, broad set of parallel activities—prioritized but with no ideas discarded—was initiated, and in the coming weeks, several critical opportunities were identified:

- R&D and fermentation PD were able to devise a screen for hardier strains, and there after identified several alternative strains
- Fermentation PD was able to identify a more robust culture medium which eliminated the ‘pouting’ behavior at lab scale
- Working with the engineering team, it was determined that the fermentation could be operated under conditions which reduce stress on the culture (Mantra #2 process development is responsible for the process—design the process as the plant exists!)

Four months later, an improved strain and fermentation process with even better performance was successfully transferred.

Much was learned from this stressful period. Our shift from the “Full Court Press” to “Lean” technology transfer support resourcing was aggressive and increased risk, and clear warning signs were ignored in part due to some hubris resulting from past technology transfer successes. Significant diversion of resources was needed to troubleshoot, creating negative knock-on effects on other projects. And the production facility missed out on the better performing fermentations in the months it took to return with a solution.

That said, for this transfer, we had assessed that there were some mitigations for a failure. Also, there were some very impactful technical lessons learned. So while on balance, more was likely gained in the long run by incurring this short-term failure, it is also clear that a net gain should not be expected to be the case in future transfers, and, therefore, we needed to be more thoughtful about balancing the risk in the technology transfer with the impact of failure and be more nuanced in the resourcing for technology transfer support.

It was clearly helpful to have a backup strain and fermentation technology to be able to rapidly deploy and minimize the impact of the initial failure. This is certainly not the case when starting up a process in a facility for the first time. The next case study describes what was learned from a challenge on such a start-up.

### Case study #2: New product start-up

The fourth fermentation product to be scaled has a unique combination of properties: it is a nearly neutrally buoyant, insoluble solid under some fermentation conditions. To develop a cost-effective, robust process to separate the product from the biomass and other insoluble solids while minimizing capital outlay at the production plant, some ingenuity was required.

The most direct approach to this was to modify the operation of one separation technique to suit the needs of the separation. This process was demonstrated to be effective at lab and pilot scale. Further, the team engaged an expert consultant, who agreed that the approach stood a reasonable chance of success, but could not guarantee it, as it had not been demonstrated previously at full scale in their experience.

Still, in discussions with the Manufacturing team, there was some concern due to this lack of precedent. Thus (since manufacturing is the customer—Mantra #1), the process development team designed a backup approach utilizing different equipment. With this approach, plant throughput would be cut in half, increasing product costs and requiring more plant time, but this backup could be implemented quickly should the novel approach fail to scale-up.

Even before start-up, there were some signs of difficulty. Tests of the full-scale equipment—now available as the previous campaign had finished—gave mixed results. This proved to foreshadow the actual process results—even with some creative modifications, the full-scale equipment did not allow the same process performance as was observed at pilot scale. Therefore, the backup approach was implemented, leading to technology transfer success, but at half the throughput.

In the after-action review, the team reflected on our technology transfer philosophy. Process development must understand the full-scale equipment and facility deeply (Mantra #2) so that they design the process for the plant as it actually exists. This technology transfer was a unique case—even without a formal risk assessment, it was clear that a backup plan would be needed. Because of that planning, the technology transfer effort ended in relative success.

Our last case study is again in the area of downstream processing. In this case, only subtle changes were being introduced for the start-up—essentially optimizing operations from the previous campaign. Yet even in this relatively low-risk scenario, a thoughtful approach to technology transfer focus was warranted.

### Case study #3: Unexpected product degradation at an intermediate hold point

In the autumn of 2018, a new campaign for our seventh fermentation molecule had started. The upstream fermentation and product isolation step were nearly identical to the previous campaign, and the downstream purification area had benefited from significant investments in new instrumentation, plant monitoring systems, additional tankage, and training for the operations team. All of this contributed to what in hindsight may have been an inappropriate degree of confidence and complacency. As is typical, the PD team supporting this campaign was multitasking—supporting the technology transfer and supporting other process development efforts at lab and pilot scale.

One of the issues that did receive significant attention during the run-up to this campaign was the pH specification on the crude product intermediate. In the prior campaign, some product losses due to microbial degradation were observed in an intermediate while it was held in inventory. At the time, the downstream processing throughput had not been fully debottlenecked to match the upstream capacity. As such, a primary concern in the new campaign was in minimizing degradation losses.

Laboratory stability studies clearly showed that holding the intermediate crude product at elevated pH inhibited microbial degradation without impacting the final product quality attributes. However, the accuracy of the inline pH control step was poor—to ensure protection from microbial contamination and limit disruption to operations, the technology transfer document specified a pH set point that was a full 1.5 units above the minimum pH required to inhibit microbial growth, allowing a fairly wide pH range of ± 1.5 at this step.

In the campaign, the purification operations started smoothly. However, as analytical data began to become available (Mantra #5), product titers were lower than expected. The technology transfer team pursued multiple lines of inquiry, quickly ruling out microbial growth and non-representative sampling. Concurrently, detailed chromatographic analysis revealed elevated concentrations of a degradation product typically not observed at this stage. pH measurements on the incoming stream confirmed that although the pH of the crude product was technically within the range allowed by the technology transfer document, it was far above the level where the vast majority of all laboratory and pilot testing had been conducted. Thus, prevention of chemical degradation became the focus.

The PD team supporting the campaign rapidly initiated stability tests that confirmed that the pH and temperature history of the crude product at manufacturing scale could indeed result in unacceptably high byproduct formation within days. After the control of pH was brought to lower levels, the pH of the crude product leaving the upstream area was decreased, and the precision of the pH control system came to be better understood.

The rapid response of the operations and PD team minimized the product losses suffered in the early stages of this campaign. However, this technology transfer experience provided important reinforcement of Mantra #3: we do not do experiments in the plant. The upper end of the allowable pH range specified in the technology transfer document was never validated at pilot, or even at lab scale. To mitigate a known risk (microbial contamination), we created a new risk (chemical degradation), which we were fortunately able to mitigate on-the-fly.

This experience was also a reminder to design the process with the equipment in mind (Mantra #2). The first step in implementing this philosophy is to properly understand the limits of the available equipment: in this case, it eventually became clear that the existing hardware was quite capable of controlling the pH of the crude product to within an acceptable pH range. Had the operations and PD team quantified these limits prior to the campaign, the early degradation losses could likely have been avoided altogether.

## Future challenges

It is our expectation that the pace at which molecules can be brought to full-scale manufacturing will continue to increase. Major investments in the automation of strain construction and advances in strain engineering prowess accumulated over the past decade will allow Amyris and others to rapidly accelerate the pace at which commercially relevant fermentation performance can be realized. For example, a decade ago it took Amyris three years to exceed 40 g/L in an amorphadiene fermentation.

In contrast, several years ago the strain engineering team was able to create a strain in nine months which made a different product at > 40 g/L and 30% of the maximum theoretical yield. Coupled with our rapid process development and efficient technology transfer, the process was delivered to full-scale manufacturing in less than twelve months from the initiation of strain engineering. Further, recently a strain making a different novel product at commercially viable performance was engineered in just four months, and the fermentation was able to achieve 50% of maximum theoretical yield in extended fermentation.

**Fig. 2:**
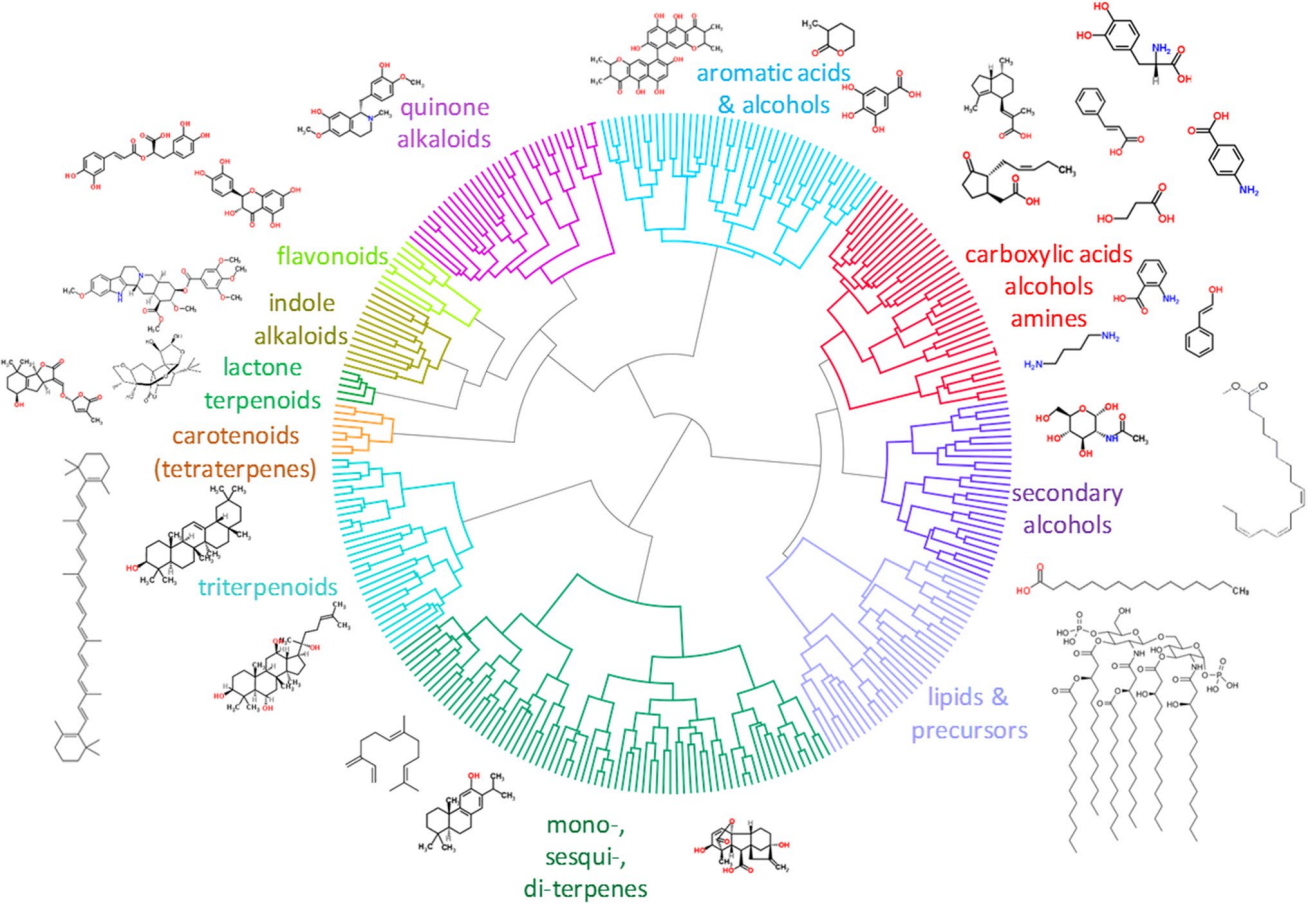
A representation of the molecules targeted by the Amyris Mgs to Kgs project (part of the DARPA-funded thousand molecules program). Strains producing hundreds of different molecules representing a huge range of small molecule space are now available for development into products. Many times more products are now available through semi-synthesis, whereby fermentation products are converted by chemical synthesis into additional products

While this will not be the case for every product pursued, the accelerated pace of strain engineering success is demanding increased speed downstream in the product development machinery. In addition, Amyris now has strains making hundreds of different molecules as a result of the “Mgs to Kgs” (M2K) program (see Fig. 2), giving us even wider access to strains making a wide variety of different natural product classes.

Therefore, in addition to optimizing the resourcing dedicated to technology transfer, organizations will require accelerated development of fermentation, DSP, and analytical processes as well as a streamlined regulatory approach to bringing products to market at a matched pace. The speed of technology maturation has other consequences for a growing organization, even impacting the pace of capital investment at manufacturing sites. For example, on a recent new product, the strain engineering and fermentation process development team was able to quadruple fermentation performance in a year, necessitating significant capital investment at the manufacturing site many months ahead of schedule.

In terms of accelerating downstream process development, it is efficient where possible to build platform development approaches that can be used for multiple molecules that fall into the same molecule classes for the steps beyond primary clarification. Likewise, when strain engineering is still in the earlier stages, constructing simulated future fermentation broths when possible may help. There is a clear need to build higher throughput tools—some unit operations such as adsorbant and solvent screening are well suited to high-throughput development approaches. Being able to automate and miniaturize some of this experimentation will not only enable coverage of more experimental space faster, but it may also enable initiation of DSP development earlier (at the 0.25 L fermentor stage for example).

Analytically, the ability to more quickly identify and measure product impurities will be increasingly important. In particular, species that are molecularly similar to the target molecule—whether they are derived from the host organism, upstream pathway intermediates, or a variant of the final product—will be the most critical.

Business success through accelerated time-to-market can be better achieved when regulatory affairs and commercial resources are integrated into the business plan. A business strategy should align the regulatory activities with the company’s product innovation and corporate success factors. The regulatory team must go beyond simply having knowledge of the regulatory requirements, but also:

- Be knowledgeable of the legislative background and intent of the regulation;
- Communicate effectively with regulatory authorities and external subject matter experts;
- Identify legitimate opportunities within the written regulation.

The regulatory strategy should utilize the collaboration of a cross-functional team, having scientific, EH&S and manufacturing, regulatory-legal and business expertise. And lastly, the team must should empowered and accountable for identifying, tracking and achieving key milestones through a project management system, periodically reporting to a well-aligned executive management team.

## Conclusions

The pace of strain engineering continues to accelerate—in many cases, it is no longer the rate limiting step to delivering products to market. Deployment of technology can be done efficiently and quickly, provided the process development systems effectively test with a scaled-down mimic of the future site, the groups involved embrace an effective technology deployment philosophy, and the technology transfer team commits the right resources to the start-up effort to match the organization’s tolerance for risk.

Building improved capabilities in downstream processing and analytics development, and smoothing the path through regulatory development, are critical capabilities to speed products to market.

## Footnotes

1 Assuming 10 day fermentations averaging 1 g/L/h volumetric productivity, with a 70% DSP yield and $15/kg pure product cost results in nearly $1350 k in delayed or lost revenue. An additional $150 k is an estimate of the facility fixed costs.

2 For example, 12 FTE × 7 months at $300 k per full time employee fully loaded.
